# Review of 500 migraine cases treated with combination of antidepressant & CBT & specific group of dietary supplement with 6 month follow up

**DOI:** 10.1186/1129-2377-14-S1-P186

**Published:** 2013-02-21

**Authors:** VK Goyal

**Affiliations:** 1 Parth Hospital, India

## 

Migraine is a condition which consumes maximum revenue of a family, state & nation & still the suffering goes on. In the world millions are suffering from Migraine .. & having pain killer medicine & suffering from their side effect like kidney failure etc Migraine sufferers may have depression deep down... but psychiatry being a stigma - very few sufferer goes for consultation to psychiatrist. History of migraine needs a long time to listen & the ability to listen ,.... which normally a psychiatrist does not have time to listen the painful past & long details without getting revenue - or fee or financially paid for - so depression goes unnoticed. Depression assessment scale -although available - but very few psychiatrist has a team work in India & most of the countries.

## Purpose

1. To see that by treating depression & adding specific group of dietary supplements - is there any effect on frequency severity & relapse? 2. Frequency of pain killer - can it be reduced & patient be saved from kidney failure?

## Methods

1. Used exclusion criteria ..... where no other cause of migraine found...all investigation within norm. 2. After assessment treated with antidepressant & CBT & specific group of dietary supplememt. In follow up video of their statement regarding frequeny was noted & use of pain killer was recorded.

## Result

1. 50 patients dropped out of the study. 2. 450 completed the study. 3. 90% of patients reported a 90% reduction in the use of pain killer. 4. 100% reported that CBT helped them. 5. 10% of patients reported a mixed picture of attacks but reported reduced severity.

## Conclusion

Migraine patient may have depression underlying which if addressed can be a useful method to reduce severity of migrane attacks. If stigma of mental health or depression can be reduced - will benefit migraine patient. Nti depressant therapy coupled with CBT & specific group of dietary supplement plays an important role in treatment of migraine .

